# Post-Processing Algorithm for Leg Electrical Impedance Imaging Integrating Boundary Attention Mechanism

**DOI:** 10.3390/s26103117

**Published:** 2026-05-15

**Authors:** Luwen Zhang, Wu Wang

**Affiliations:** Department of Electrical Engineering, Guizhou University, Guiyang 550000, China; 15584215755@163.com

**Keywords:** electrical impedance imaging, boundary attention mechanism, generative adversarial networks, leg tissue imaging

## Abstract

In impedance imaging, the incompatibility and nonlinearity of the inverse problem lead to problems such as blurred boundaries and severe artifacts in the reconstructed images, making it difficult to meet the requirements for precise identification of multi-layer tissue structures in the legs. To this end, this paper proposes a post-processing algorithm for leg EIT that integrates the boundary attention mechanism, with a Wasserstein generative adversarial network as the training framework, cyclic residual U-Net as the generator, and the boundary attention module embedded in the RecurrentBlock. This leads to adaptive enhancement of the ability to extract organizational boundary features through a three-path fusion of spatial attention, channel attention, and learnable Laplacian edge enhancement. A leg anatomy prior constraint loss function was designed, integrating six constraints—pixel loss, edge loss, hierarchical tissue constraint, total variation regularization, structural similarity loss, and histogram matching—to guide the reconstruction results to conform to the multi-layered tissue structure features of the leg. A simulation dataset of leg sections containing multiple tissues such as skin, fat, muscle, bone, blood vessels, and nerves was constructed, and the pre-reconstructed images were obtained using the hybrid total variation regularization algorithm as the network input. The simulation results show that, under noise-free and different signal-to-noise ratio conditions, the proposed BAM-R2UNet algorithm achieves the best performance in RMSE, SSIM and PSNR metrics compared with HTV, DnCNN and standard U-Net algorithms, can remove artifacts, accurately restore the boundary and conductivity distribution of leg tissues, and has stronger anti-noise robustness.

## 1. Introduction

Electrical impedance tomography (EIT) is a non-destructive testing technique [1] that uses a reconstruction algorithm to obtain an image of the internal conductivity distribution by applying an excitation current at the boundaries of the measurement domain and collecting boundary voltages. This technique has the advantages of being non-invasive, radiation-free, low-cost, and capable of real-time imaging, and has broad application prospects in fields such as biomedicine [2], agricultural geology [3], and industrial inspection [4]. Leg EIT imaging has potential future clinical value for the early detection of deep vein thrombosis, tissue edema, tumors and other lesions of the lower extremities. The single EIT inverse problem is highly ill-defined and nonlinear [5], resulting in widespread problems such as low spatial resolution, blurred boundaries and severe artifacts in reconstructed images. For complex anatomical structures such as the leg, which contains multiple layers of tissue including skin, fat, muscle, bone, and blood vessels, traditional reconstruction algorithms often have difficulty accurately identifying the boundaries and conductivity distribution of each layer of tissue, limiting the application of EIT technology in leg clinical diagnosis.

In recent years, deep learning has been widely applied in the post-processing of EIT images, mainly using an image-to-image strategy to optimize pre-reconstructed images. Wang et al. [6] proposed a DNCNN-based post-processing method that removes reconstruction artifacts through residual learning, but has limited ability to preserve tissue boundaries. Li et al. [7] proposed a post-processing algorithm based on WGAN-gp, which uses the adversarial training mechanism of the generative adversarial network to optimize the pre-reconstructed image and reduce the artifacts of the target object. Ye et al. [8] employed the WGAN-R2UNet algorithm, using recurrent blocks instead of traditional convolutional layers to enhance feature capture capabilities and achieved good imaging results under various noise conditions. Yuan et al. [9] proposed the S-PNet algorithm, which uses a pyramid pooling structure to extract multi-scale features for the segmentation of adhesion pressure points. Ye et al. [10] established a multi-branch one-dimensional convolutional neural network model, which enhanced the anti-noise ability of traditional algorithms. However, the aforementioned methods mostly target simple geometric shapes (circles, squares, triangles, etc.) and do not utilize prior knowledge of specific anatomical structures. For imaging objects with well-defined anatomical stratification structures such as legs, the lack of targeted boundary enhancement mechanisms and anatomical constraints leads to insufficient ability to preserve tissue boundaries.

To address these issues, this paper proposes a post-processing algorithm for leg EIT that integrates the boundary attention mechanism. The main innovations include: (1) embedding the boundary attention module (BAM) in the RecurrentBlock of R2UNet, which adaptively enhances the extraction ability of tissue boundary features such as skin–fat, fat–muscle, and muscle–bone through a three-path fusion mechanism of spatial attention, channel attention, and learnable Laplacian edge enhancement; (2) designing a leg anatomy prior constraint loss function, fusing six constraint-guided networks to output reconstruction results that conform to the multi-layered tissue structure features of the leg; (3) constructing a leg-specific multi-tissue simulation dataset to systematically verify the robustness and noise resistance of the algorithm.

## 2. Related Theories

### 2.1. HTV Pre-Reconstruction Algorithm

The hybrid total variation (HTV) algorithm [11] is an EIT imaging algorithm proposed by Zuo Congli, combining the L2 norm Noser (Newton’s one-step error reconstruction) penalty function term and the L1 penalty function term. The solution framework is derived based on the primal dual interior point method (PDIPM). The optimization objective of the HTV algorithm is expressed as

(1) $$\hat{\sigma }=argmin\|V-F(\sigma ){\|}^{2}+\alpha \|L\sigma \|2^{2}+\beta \|\sigma \|1$$

In the formula, F(σ) is the positive problem operator; V is the measurement voltage vector; L is the regularization matrix; and α and β are regularization parameters.

Compared with the Tikhonov regularization algorithm, the algorithm has significantly improved image quality and can better preserve the edge features of the image.

However, the HTV algorithm still has problems such as boundary blurring, artifacts, and shape and size recognition errors in EIT imaging, especially in the precise identification of the multi-layered tissue structure of the legs. In this paper, HTV pre-reconstructed images are used as input for the deep learning post-processing network, and the pre-reconstructed images are optimized using the BAM-R2UNet algorithm.

### 2.2. WGAN Algorithm

A generative adversarial network (GAN) [12] consists of a generator G and a discriminator D. The generator G takes input x and attempts to generate a synthetic data sample G(x) similar to the real data sample y; discriminator D takes G(x) as well as y and tries to distinguish them. The generator and the discriminator are trained alternately until the discriminator can no longer distinguish between synthetic data samples and real data samples, at which point the generator can produce realistic synthetic data.

GAN models have problems such as vanishing gradients, training instability, and pattern crashes. To address these issues, Arjovsky et al. [13] proposed a generative adversarial network (WGAN) based on Wasserstein distance optimization in 2017. For the two probability distributions Pr and Pg, the Wasserstein distance is defined as

(2) $$W(P_{r},P_{g})=inf{inf}_{\gamma \in \Pi (P_{R},P_{G})}E_{(x,y)\sim \gamma }[\left\|x-y\right\|]$$

In the formula, γ is the joint distribution of Pr and Pg combinations; ∏(Pr, Pg) is the set of all possible γ; G(x) is the generated sample; y is a real sample; and ‖G(x) − y‖ is the distance between the samples. The Wasserstein distance, compared to the KL divergence and JS divergence, provides meaningful gradient information even when the two distributions do not overlap, thus solving the problem of training instability. Based on this, Gulrajani et al. [14] proposed the WGAN-gp model with a gradient penalty (GP), which optimizes the modeling ability of the discriminator by adding a gradient penalty term to the discriminator’s objective function to satisfy the Lipschitz constraint. The gradient penalty term is defined as

(3) GP=λEy^∼Py^[Gx−y]

In the formula, λ is the weight coefficient that controls the gradient penalty; y^ is the random interpolation between the real sample y and the generated sample G(x); D(y^) is the discriminator’s output to the interpolated sample y^; and ∇y^D(y^) is the gradient of D(y^)y^. This paper optimizes the generator network based on the WGAN-gp model, using the R2UNet architecture that fuses the boundary attention mechanism as the generator.

### 2.3. Recurrent Residual U-Net (R2UNet)

Recurrent Residual U-Net (R2UNet) is an improved architecture based on the classic U-Net encoder–decoder structure. It integrates recurrent convolution blocks and residual connections to enhance the ability of multi-scale feature extraction and detail preservation. The recurrent block accumulates feature information through multiple time-step iterations, which strengthens the capture of context and local details. The residual connection alleviates the gradient vanishing problem in deep network training and improves training stability and convergence speed. Compared with standard U-Net, R2UNet can better recover structural details and edge information, making it more suitable for EIT image reconstruction tasks with blurred boundaries and complex tissue distributions.

## 3. A Post-Processing Algorithm Integrating the Boundary Attention Mechanism

The direct correspondence between the key problems in leg EIT reconstruction and the targeted solutions of each component in the proposed method is further clarified. The severe blurring of tissue boundaries is addressed by the boundary attention module (BAM), in which the learnable Laplacian edge enhancement branch, spatial attention, and channel attention adaptively enhance boundary feature extraction and sharpen tissue edges. Widespread reconstruction artifacts are suppressed by the WGAN-GP adversarial training framework and the total variation regularization in the loss function, which eliminate false structures and high-frequency noise while preserving structural details. The difficulty in identifying multi-layer leg tissue structures is solved by the leg anatomy prior constraint loss, especially the layered tissue constraint term, which guides the network to output reconstruction results consistent with the concentric multi-layer anatomical characteristics of leg tissues. The cyclic residual structure of R2UNet further strengthens feature representation and improves the stability and accuracy of conductivity reconstruction.

**Figure 1:**
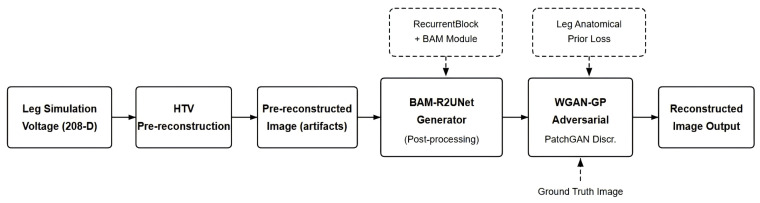
Flowchart of leg EIT post-processing algorithm fused with boundary attention mechanism.

The overall process of the post-processing algorithm based on BAM-R2UNet is shown in Figure 1. First, based on the boundary voltage data of the leg section, a pre-reconstructed image of the electrical impedance distribution with artifacts and boundary blurring is obtained through the HTV algorithm. The pre-reconstructed images are used as input to the BAM-R2UNet generator, while the ideal simulated conductivity images are used as real labels, and the generator and discriminator are alternately optimized through the WGAN adversarial training framework. After training, the generator can map HTV pre-reconstructed images to high-quality conductivity reconstructed images, remove artifacts and enhance the clarity of tissue boundaries.

### 3.1. Architecture Design of the BAM-R2UNet Generator

**Figure 2:**
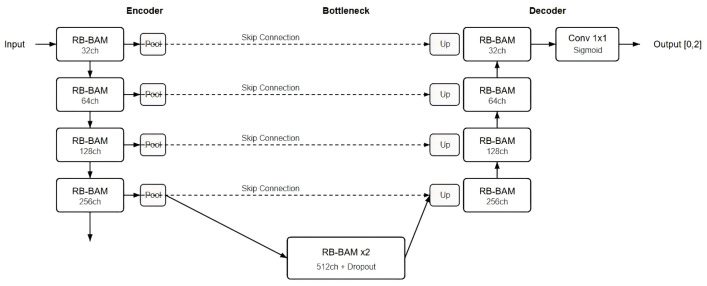
BAM-R2UNet network structure.

The generator of the algorithm in this paper uses a cyclic residual U-Net (BAM-R2UNet) that fuses the boundary attention mechanism, and its overall structure is shown in Figure 2. The main body of the network is a symmetric architecture of an encoder–bottleneck layer and decoder. The encoder consists of four downsampling modules, each of which first projects the channel through a 1 × 1 convolutional layer, then extracts features through two cascaded RecurrentBlock-BAM convolutional blocks, and finally halves the space size through a Max pooling layer. From top to bottom, the number of channels in each layer is 32, 64, 128, and 256 respectively. The spatial size of the feature map decreases gradually, while the degree of feature abstraction increases gradually. The bottleneck layer consists of a 1 × 1 channel projection and two RecurrentBlock-BAM convolutional blocks, which have 512 channels and are regularized using Dropout (at a ratio of 0.1).

The decoder section is symmetrically designed with the encoder and gradually restores the spatial resolution of the image through four upsampling modules. Each upsampling module first doubles the feature map and halves the number of channels through ConvTranspose2d, then concatenates and fuses it with the skip connection features of the corresponding layer of the encoder, and after 1 × 1 convolutional projection, it is sent to two RecurrentBlock-BAM convolutional blocks for feature processing. Skip connections introduce the multi-level feature information saved in the encoder into the decoder, helping to restore the spatial details of the reconstructed image. At the last layer of the decoder, the number of channels is reduced to one through 3 × 3 convolutional layers and 1 × 1 convolutional layers, and the output is limited to a conductivity range of [0, 2] through the Sigmoid activation function and scaling operations.

**Figure 3:**
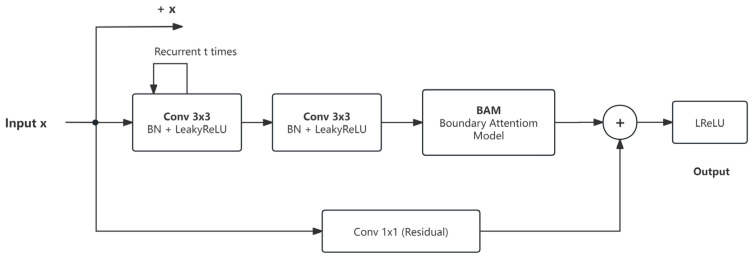
RecurrentBlock-BAM convolutional block structure.

The RecurrentBlock-BAM convolutional block is the basic building block of the network in this paper, and its structure is shown in Figure 3. The module employs the boundary attention module (BAM) on top of R2UNet’s RecurrentBlock. The RecurrentBlock contains two consecutive 3 × 3 convolutional layers, each followed by BatchNorm batch normalization and a LeakyReLU activation function (with a negative slope of 0.2). The core feature is the accumulation of features over multiple time steps through loop connections. Let the input be x and the number of loops be t, then the circular forward propagation process of the first convolutional layer is

(4) $$h^{0}={Conv}_{1}(x)$$

(5) $$h^{i}={Conv}_{1}(h^{i-1}+x),i=1,\ldots ,t-1$$

In the formula, Conv_1_ is the first convolutional layer. The output after cycling t times is processed by the second convolutional layer and then sent to the boundary attention module (BAM). After mapping the input x to the same number of channels as the output through 1 × 1 residual convolution, adding it to the BAM output and activating it with LeakyReLU, residual learning is achieved. This cyclic residual structure accumulates features over multiple time steps, providing richer feature representations, while residual connections alleviate vanishing gradients in deep networks. In this paper, the number of cycles t = 2 is set.

**Figure 4:**
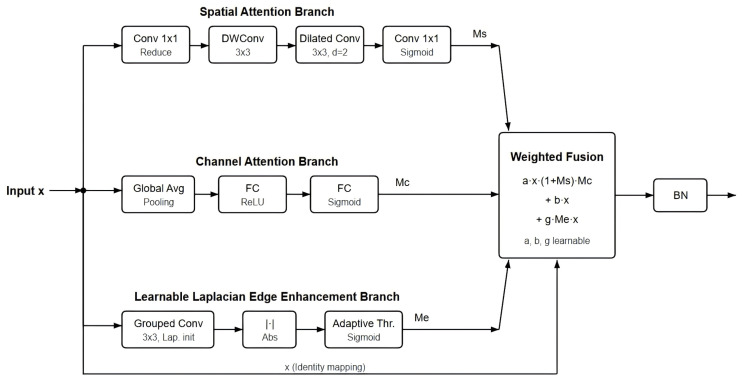
Boundary attention module (BAM) structure.

The boundary attention module (BAM) is the core innovation of the algorithm in this paper, and its structure is shown in Figure 4. The module is designed to adaptively enhance the feature response of the organization’s boundary region and consists of three parallel branches:

(1) Spatial attention branches: Branches are used to capture differences in importance at different spatial locations in an image. Specifically, the input features are first dimensionally reduced through 1 × 1 convolution (down to channels/reduction; reduction = 8), and then multi-scale spatial features under different receptive fields are extracted successively through 3 × 3 depth-separable convolution and 3 × 3 dilated convolution (expansion rate of 2). BatchNorm and ReLU activation functions are attached to each convolutional layer. Finally, the number of channels is compressed to one by 1 × 1 convolution, and the spatial attention weight map Ms is output by the Sigmoid function.
(2) Channel attention branches: Branches are used to model dependencies between different feature channels. First, the spatial information of each channel is compressed into a single scalar value through global average pooling, then the information exchange and compression–excitation operation between channels are carried out through two layers of fully connected networks (with ReLU activation in between), and finally the channel attention weight vector Mc is output through the Sigmoid function.
(3) Learnable Laplacian edge enhancement branches: Branches are key designs that distinguish BAMs from conventional attention mechanisms and are specifically designed to enhance feature responses at organizational boundaries. Implemented with grouped convolution, each channel performs the convolution operation independently, with the initial weights set to the standard Laplacian kernel. Unlike fixed edge detection operators, the weights of the convolution kernels can be automatically updated through backpropagation during training, enabling the network to adaptively learn the edge detection features that best suit the characteristics of EIT images. After taking the absolute value of the edge response, the edge weight map Me is generated by the adaptive threshold (based on the mean of the feature map) and the Sigmoid function.

The outputs of the three branches are weighted and fused by learnable parameters α, β, and γ, he reconstructed image

(6) $$BAM(x)=BN(\alpha \cdot x\cdot (1+M_{s})\cdot M_{c}+\beta \cdot x+\gamma \cdot M_{e}\cdot x)$$

In the formula, α, β, and γ are initialized to 0.5, 0.3, and 0.2, respectively, and are automatically optimized through backpropagation during training; BN is the batch normalization layer. The first α·x·(1 + Ms)·Mc achieves joint modulation of spatial and channel attention, allowing the network to focus on both important spatial locations and feature channels simultaneously. The second β·x is an identity mapping that retains the original feature information to ensure training stability. The third item, γ·Me·x, incorporates edge-enhanced information into the feature representation and adaptively reinforces the response of the tissue boundary region. This three-path fusion strategy enables the network to specifically enhance the representation of organizational boundaries while maintaining the integrity of the overall features.

The discriminator adopts the PatchGAN architecture. Its design concept is to make authenticity judgments on local areas (patches) of the image rather than providing a single true and false scalar for the entire image. The discriminator consists of five convolutional layers with a kernel size of 4 × 4. The first four layers have a step size of 2 (the last layer has a step size of 1), followed by InstanceNorm normalization (except for the first layer) and LeakyReLU activation function (with a negative slope of 0.2). The number of channels increases gradually from 64 for the first layer to 512 for the fourth layer. Ultimately, the convolutional layer outputs a single-channel feature map, where each element represents the authenticity judgment of a local area within the receptive field range. The discriminator’s input consists of two channels: the conditional image (HTV pre-reconstructed image) and the stitching of the target image in the channel dimension. This conditional discriminator design enables the discriminator to focus not only on the authenticity of the generated image but also on its correspondence with the input HTV image.

### 3.2. Leg Anatomy Prior Constraint Loss Function

EIT post-processing algorithms typically constrain the network output using only pixel-level losses, such as L1 or L2 losses, ignoring the structural features of the imaging object itself. The cross-section of the leg has a distinct concentric multi-layer tissue distribution: from the outside to inside it is skin, subcutaneous fat, and muscle, and in the central area there are bone, blood vessels, and nerves, and the electrical conductivity of each layer of tissue has characteristic differences. Based on this anatomical prior knowledge, this paper designs a composite loss function that integrates six constraints:

(7) $$L_{total}=\lambda_{pix}L_{pix}+\lambda_{edge}L_{edge}+\lambda_{layer}L_{layer}+\lambda_{tv}L_{tv}+\lambda_{ssim}L_{ssim}+\lambda_{hist}L_{hist}$$

The definitions and physical meanings of each loss are as follows.

(1) Pixel loss Lpix: A weighted combination of L1 loss and L2 loss is used to constrain the accuracy of global conductivity values. L1 loss is insensitive to outliers and helps maintain the overall structure of the image; L2 loss has a stronger penalization effect on large errors, which helps to reduce reconstruction bias:

(8) Lpix=‖y^−y‖1+0.5‖y^−y‖22

where y^ is the predicted output of the network; y is the real conductivity image.

(2) Edge loss Ledge: Using the Sobel operator to extract the gradients of the predicted image and the real image in the horizontal and vertical directions respectively, calculate the L1 distance between the gradient magnitude maps for constraining the accuracy of the organizational boundary:

(9) Ledge=‖E(y^)−E(y)‖1

In the formula, E(·) represents the Sobel edge detection operator, which calculates the gradient magnitudes after the image gradients in the x and y directions. The loss term prompts the network to focus during training on regions where conductivity changes occur in the image, that is, tissue boundary positions.

(3) Layered tissue constraint loss Llayer: In the loss function, the concentric multi-layer tissue distribution characteristics of the leg cross-section are utilized. Based on the distance from the pixel to the center of the image, the image domain is divided into four annular regions—the skin layer, the fat layer, the muscle layer, and the core layer—and the average conductivity of each region is constrained to be consistent with the true value:

(10) Llayer=∑k=14|mean(y^⋅Mk)−mean(y⋅Mk)|

In the formula, Mk is the mask of the KTH layer of tissue, the skin layer mask corresponds to the annular area (R is the radius of the leg section) at a distance from the center, the fat layer is R-10 to R-3, the muscle layer is within R-10, and the core layer is within 0.35R. The constraint ensures that the conductivity distribution of the reconstructed image at each tissue level is statistically consistent with the true value.

(4) Total variation regularization loss Ltv: By calculating the L1 norm of the conductivity difference between adjacent pixels, high-frequency noise and unnatural pixel-level fluctuations are suppressed in the reconstructed image while preserving the image edges:

(11) Ltv=∑i,j(|y^i,j−y^i+1,j|+|y^i,j−y^i,j+1|)

(5) SSIM loss Lssim: This is constructed using 1 minus structural similarity, maintaining consistency in the three dimensions of luminance, contrast, and structure between the reconstructed image and the real image:

(12) $$L_{ssim}=1-SSIM(ŷ,y)$$

The SSIM is calculated using a Gaussian weighted window with a size of 11 and a standard deviation of 1.5.

(6) Histogram matching loss Lhist: The L1 distance is calculated after all pixel values of the predicted image and the real image are sorted separately, and the gray value distribution of the two images is constrained to be consistent:

(13) $$L_{hist}=\|sort(ŷ)-sort(y){\|}_{1}$$

The weighting coefficients for each item take into account the magnitude differences and relative importance of different constraints, specifically: λpix = 100, λedge = 30, λlayer = 20, λtv = 5, λssim = 10, and λhist = 2. Pixel loss weights are maximized to ensure global reconstruction accuracy. Edge and layering constraints come second to enhance the preservation of structural features. Total variation and histogram matching are used as auxiliary constraints for regularization.

The total loss of the generator is the sum of the WGAN adversarial loss and the aforementioned anatomical prior constraint loss:

(14) $$L_{G}=-E[D(x,G(x))]+L_{total}$$

The loss function of the discriminator includes the Wasserstein distance and the gradient penalty term:

(15) $$L_{D}=E[D(x,G(x))]-E[D(x,y)]+\lambda_{gp}\cdot GP$$

In the formula, x is the HTV pre-reconstructed image; G(x) is the generator output; y is the real conductivity image; and λgp = 10 is the gradient penalty coefficient. During the training process, train the discriminator n_critic = 5 times for each training step, and then train the generator once to ensure that the discriminator has sufficient discriminative power to provide the gradient signal for the generator.

This anatomy-driven loss ensures the network learns EIT-specific conductivity mapping aligned with leg tissue structure, instead of performing blind image restoration.

### 3.3. Performance Evaluation Metrics

To objectively evaluate the quality of EIT post-processed images, root mean square error (RMSE), structural similarity index measure (SSIM), and peak signal-to-noise ratio (PSNR) were selected as evaluation metrics.

RMSE measures the size of the pixel-level deviation between the predicted image and the real image, and the smaller the value, the higher the reconstruction accuracy. The definition of RMSE is

(16) $$RMSE=\sqrt{\frac{1}{N}\sum_{i=1}^{H}\sum_{j=1}^{W}[S(i,j)-G(i,j){]}^{2}}$$

In the formula, H and W are respectively the height and width of the image; N = H × W is the total number of pixels; and S(i,j) and G(i,j) are the pixel values of the real image and the reconstructed image at the corresponding positions (i,j), respectively.

SSIM evaluates the similarity of two images by taking into account three dimensions: luminance, contrast, and structure. The closer the value is to 1, the more similar the reconstructed image is to the real image. The SSIM is defined as

(17) $$SSIM(X,Y)=\frac{\left(2\mu_{X}\mu_{Y}+C_{1})(2\sigma_{XY}+C_{2}\right)}{\left(\mu_{X}^{2}+\mu_{Y}^{2}+C_{1})(\sigma_{X}^{2}+\sigma_{Y}^{2}+C_{2}\right)}$$

In the formula, $\mu_{X}$ and $\mu_{Y}$ are the averages of the two images respectively; $\sigma_{X}$ and $\sigma_{Y}$ are standard deviations respectively; $\sigma_{XY}$ is covariance; and C_1_ = 6.5025 and C_2_ = 58.5225 are stability constants that prevent the denominator from being zero.

The PSNR measures the level of the signal-to-noise ratio of the reconstructed image relative to the real image, and the larger the value, the better the reconstruction quality. The definition of PSNR is

(18) $$PSNR=10\cdot lg\frac{{MAX}^{2}}{MSE}$$

In the formula, MAX is the maximum value of the image pixel (the conductivity range in this paper is [0, 2], so MAX = 2.0); MSE is the mean square error.

## 4. Simulation Experiments and Result Analysis

### 4.1. Establishment of the Leg Simulation Dataset

**Figure 5:**
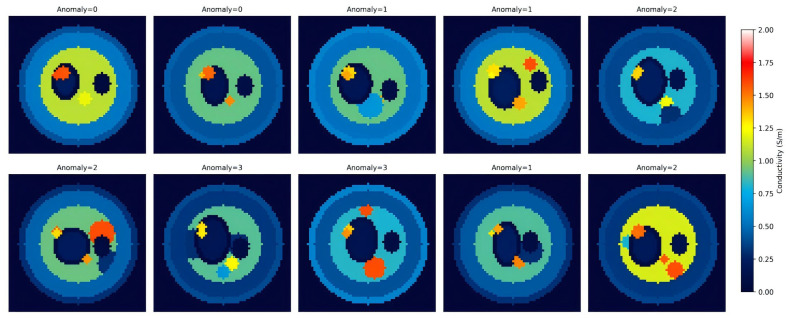
Sample of the leg section simulation dataset.

To verify the performance of the algorithm in leg EIT imaging, this paper constructs a multi-tissue simulation dataset of leg cross-sections. A parametric simulation model was established with reference to the anatomical structure of the human leg cross-section and the conductivity reference values of each tissue at 1 kHz. The model contains the following tissue layers: (1) the skin layer, located on the outermost layer, with a thickness of 1–3 pixels and an electrical conductivity of approximately 0.30–0.60 S/m; (2) the subcutaneous fat layer, located on the inner side of the skin, with a thickness of 5–10 pixels and an electrical conductivity of approximately 0.30–0.55 S/m; (3) the muscle layer, occupying the main area of the cross-section, with an electrical conductivity of approximately 0.80–1.20 S/m; (4) the tibia, located at the eccentric position of the cross-section and composed of cortical bone (conductivity 0.05–0.15 S/m) and cancellous bone (conductivity 0.10–0.25 S/m); (5) the fibula, located on the outer side of the section and composed of cortical bone; (6) blood vessels, distributed in the muscular layer, with an electrical conductivity of about 1.20–1.60 S/m; (7) nerve bundles, with an electrical conductivity of about 0.08–0.20 S/m. In addition, 0–3 pathological abnormalities were randomly placed in the muscle area, including edema (about 0.80 S/m), tumors (about 0.30 S/m), hematoma (about 0.15 S/m), and foreign bodies, etc., to simulate various lesions that may occur in actual clinical scenarios. Random perturbations were added to the geometric parameters (location, size, angle, and shape) and electrical conductivity values of each tissue within the reference range to enhance the diversity of the dataset and the generalization ability of the model. Some samples of the dataset are shown in Figure 5.

The positive problem solution uses a 16-electrode model, and in the data acquisition mode of adjacent excitation and adjacent measurement, the 208-dimensional boundary voltage vector is calculated using a sensitivity matrix method. To enhance the model’s anti-noise robustness, Gaussian white noise is added to the training samples in an alternating manner of no noise and SNR = 30, 45, and 55 dB. The expression for the signal-to-noise ratio (SNR) is

(19) $$SNR=10lg\frac{RMS(V)}{RMS(N)}$$

In the formula, RMS represents solving the root mean square; V is the original boundary voltage signal; and N is the noise signal. After obtaining the boundary voltage, pre-reconstruction is performed through the HTV algorithm to obtain pre-reconstructed images with artifacts as the network input, and the corresponding ideal conductivity distribution is used as the training label.

A total of 4800 simulation samples were generated, with image sizes of 64 × 64 pixels and conductivity values normalized to the range of [0, 2]. The dataset was randomly divided into a training set (3840 sets), a validation set (480 sets), and a test set (480 sets) in proportions of 80%, 10%, and 10%, with no overlap among them. The training set used data augmentation strategies, including random rotations (0°, 90°, 180°, and 270°), random horizontal flips, and small noise perturbations, to enrich the diversity of the training samples.

### 4.2. Model Training

Model training was performed on NVIDIA Gpus based on the PyTorch 2.1 deep learning framework. The GPUs used were manufactured by NVIDIA Corporation (Santa Clara, CA, USA). The main training parameters were set as shown in Table 1.

**Figure 6:**
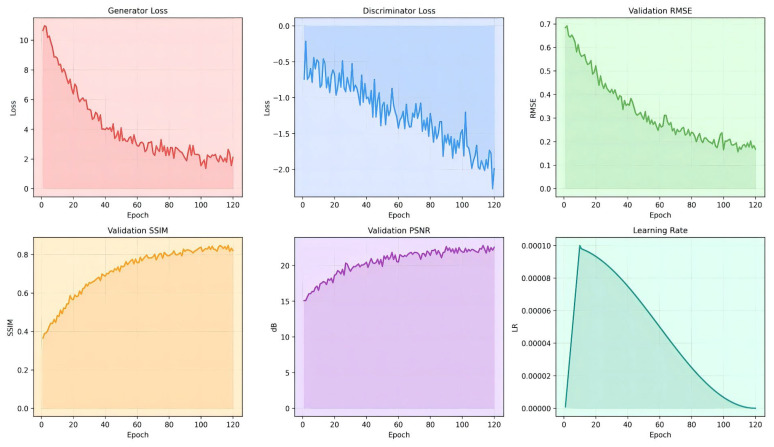
BAM-R2UNet training process curve.

Adversarial training was conducted using the WGAN-GP strategy. Generator parameters were initialized with Kaiming, and discriminator parameters were initialized with normal distribution (mean 0; standard deviation 0.02). Gradient clipping (with a maximum norm of 1.0) was used during training to prevent gradient explosion, and a cosine annealing learning rate scheduling strategy was employed to smoothly reduce the learning rate from the initial value to 1 × 10^−6^. The loss variation and validation set metric curves during training are shown in Figure 6. It can be observed that the generator loss and discriminator loss gradually stabilize during training, while the RMSE of the validation set continues to decline and the SSIM gradually increases and converges, indicating that the training process is stable. The optimal model parameters based on the SSIM value of the validation set were selected for subsequent test evaluation. The number of trainable parameters for the BAM-R2UNet generator was approximately 12.6 M, and for the PatchGAN discriminator, it was approximately 2.8 M.

### 4.3. Reconstruction Results Under Noise-Free Conditions

The image reconstruction performance of the BAM-R2UNet model was validated using test set samples and compared with the HTV algorithm, DnCNN model [15], and standard U-Net model. Among them, the DnCNN is a 17-layer depth-denoised convolutional network with a residual learning strategy [16,17]; U-Net is a standard encoder–decoder architecture that does not include BAMs and loop structures [18,19]. The control models were trained using the same training set and leg anatomy prior constraint loss function to ensure fairness in the comparison.

**Figure 7:**
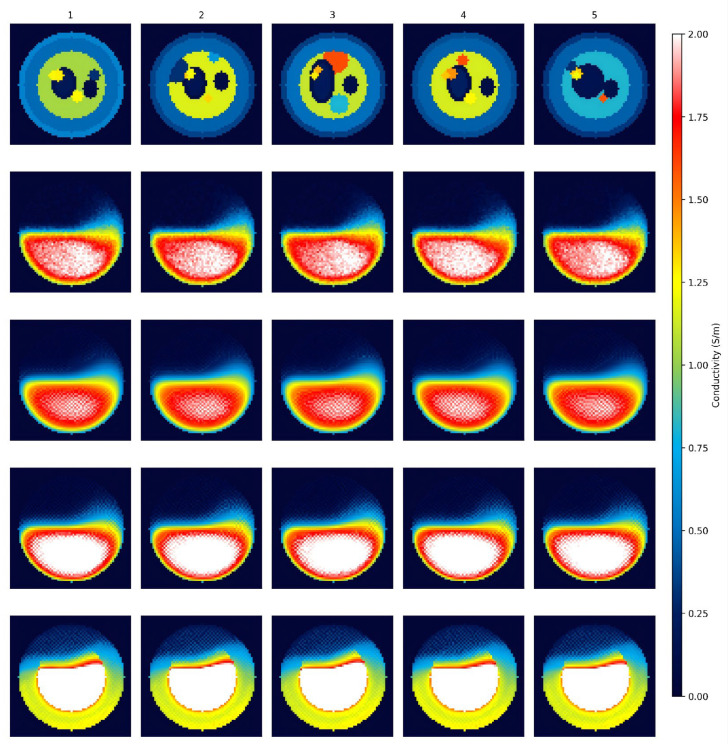
Reconstruction results of different algorithms under noise-free conditions. The columns from left to right represent 5 different typical test samples. Rows correspond to: ground truth, HTV, DnCNN, U-Net, and the proposed BAM-R2UNet algorithm. The color bar indicates the electrical conductivity distribution (unit: S/m).

Figure 7 shows the reconstruction results of each algorithm under noise-free conditions. As can be seen from Figure 7, the boundaries of the HTV pre-reconstructed image are blurred, the conductivity transitions between the layers of tissue are not clear enough, and there are obvious artifacts. The DnCNN suppressed artifacts to some extent through residual learning, but the tissue boundaries are still not sharp enough, and there are deviations in the morphology and conductivity values of some abnormal targets. The standard U-Net performs better than DnCNN in reconstruction, but has limited ability to restore structures such as tiny blood vessels and nerves. In the reconstructed images of BAM-R2UNet, the layers of tissue structure are distinct, the boundaries of skin, fat and muscle are clearly distinguishable, structures such as bones and blood vessels are well restored, and the morphology, position and conductivity distribution of abnormal targets can be accurately reconstructed.

Table 2 presents the performance parameters of the images reconstructed by the four algorithms under noise-free conditions. BAM-R2UNet achieved the best values in all three metrics: the lowest RMSE, the highest SSIM, and the highest PSNR. Compared with the HTV algorithm, BAM-R2UNet’s RMSE decreased by 60.1%, SSIM increased by 56.7%, and PSNR increased by 7.7 dB. Compared with DnCNN and U-Net, there were also significant improvements in various indicators, quantitatively confirming that BAM-R2UNet had the best reconstruction effect.

### 4.4. Reconstruction Results Under Noisy Conditions

**Figure 8:**
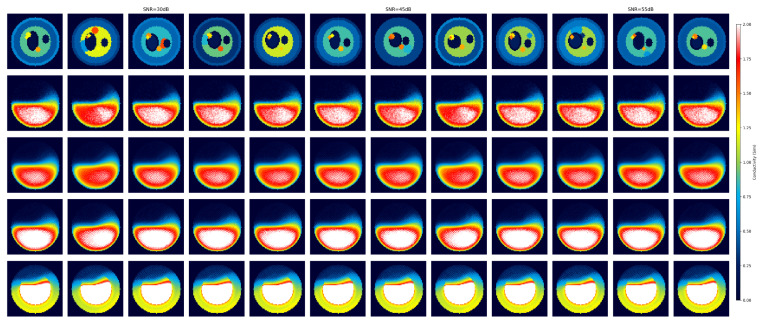
Reconstruction results under different SNR levels (30 dB, 45 dB, and 55 dB). From left to right, the entire columns are divided into three groups corresponding to 30 dB, 45 dB, and 55 dB noise conditions, respectively. Rows sequentially correspond to the ground truth, HTV, DnCNN, U-Net, and the proposed BAM-R2UNet algorithm. The color bar indicates the electrical conductivity distribution (unit: S/m).

To verify the anti-noise performance of the algorithm, reconstruction comparison experiments were conducted at three noise levels of SNR = 30, 45, and 55 dB. Figure 8 shows the reconstructed images of each algorithm under SNR = 30 dB (strong noise). At this noise level, the imaging quality of the HTV algorithm is severely reduced, with a large number of artifacts making the tissue structure almost indistinguishable. Although the DnCNN and U-Net can partially suppress noise, there are still significant errors in target recognition, and multiple abnormal targets are missed or misjudged. BAM-R2UNet can still clearly restore the basic structure of the leg tissue under strong noise conditions, and the positions and shapes of the main abnormal targets can be correctly identified. At SNR = 45 dB and 55 dB, the reconstruction effect of BAM-R2UNet is further improved, and it is able to accurately identify the size, shape, position and conductivity value of the target.

Table 3 presents the average RMSE, average SSIM and average PSNR values of the images reconstructed by the four algorithms under different SNR conditions. Under all noise conditions, BAM-R2UNet achieved the lowest RMSE value, the highest SSIM value and the highest PSNR value. Especially under strong noise conditions with SNR = 30 dB, the SSIM value of BAM-R2UNet was approximately 114% higher than that of HTV, approximately 79% higher than that of the DnCNN, and approximately 61% higher than that of U-Net, indicating that BAM-R2UNet has significantly superior feature extraction capabilities in strong noise environments. From SNR = 55 dB to SNR = 30 dB, the SSIM of BAM-R2UNet decreased from 0.8362 to 0.7534, a reduction of only 9.9%, while HTV decreased from 0.5312 to 0.3521, a reduction of 33.7%, further confirming that BAM-R2UNet has stronger anti-noise robustness.

**Figure 9:**
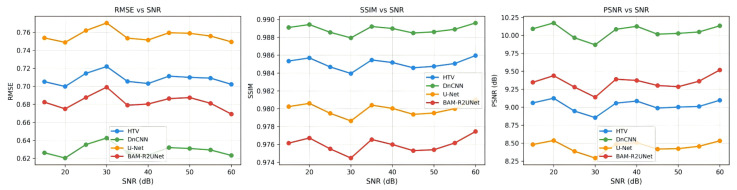
Comparison curves of the robustness of the PSNR with SNR in each algorithm.

Figure 9 shows the comparison curves of the robustness of PSNR varying with SNR for each algorithm. It can be observed that the PSNR of all algorithms decreases as noise increases (SNR decreases), but BAM-R2UNet has the smallest decline and remains leading throughout the SNR range. The HTV algorithm is most sensitive to noise and its performance deteriorates sharply under low-SNR conditions. The DnCNN and U-Net have moderate anti-noise capabilities. The superior anti-noise performance of BAM-R2UNET is attributed to the adaptive enhancement ability of the BAM to the tissue boundary and the structural guidance of the leg anatomy prior constraint loss function to the reconstruction results.

### 4.5. Ablation Experiment Versus BAM Visualization Analysis

Ablation experiments were designed to verify the contribution of the BAM and the cyclic residual structure to the performance of the algorithm. With SNR = 45 dB as the evaluation condition, the performance of four configurations—HTV (no post-processing), standard U-Net (no BAM, and no loop structure), DnCNN, and complete BAM-R2UNet—was compared, and the results are shown in Table 4.

As shown in Table 4, even the simplest standard U-Net post-processing can significantly improve the quality of HTV pre-reconstruction (RMSE reduced by 29.9%, and SSIM increased by 29.4%), indicating that the deep learning-based post-processing strategy is the best. The complete BAM-R2UNet further reduced RMSE by 42.8%, increased SSIM by 21.7%, and improved PSNR by 4.2 dB compared to the standard U-Net baseline. This significant improvement comes from two aspects: one is the multi-step feature accumulation ability of the RecurrentBlock, which enables the network to capture deeper abstract features; second, the adaptive enhancement of the BAM to the organizational boundary gives the network higher resolution in the boundary area.

**Figure 10:**
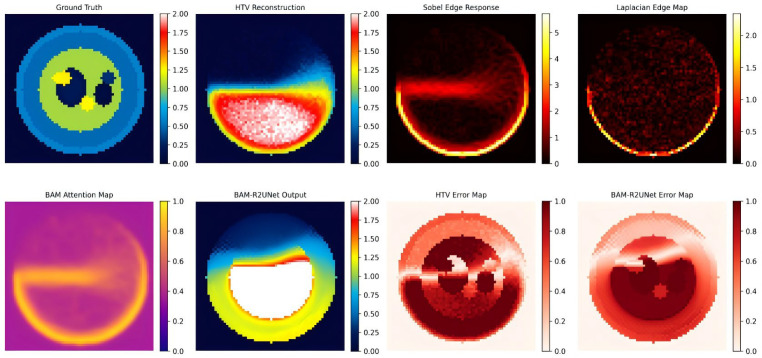
Visual analysis of BAM feature maps.

To visually demonstrate how the BAM works, Figure 10 presents the visualization results of the BAM feature map. From left to right in the top row are the true conductivity map, the HTV pre-reconstruction map, the Sobel edge response map, and the Laplacian boundary map. The lower row consists of the BAM attention weight map, the BAM-R2UNet output map, the HTV error map, and the BAM-R2UNet error map. It is clearly observable from the BAM attention weight map that there are significant high response values (bright areas) at the boundaries of tissues such as skin–fat, fat–muscle, and muscle–bone, while the responses are lower in the uniform areas within the tissues. This suggests that the BAM is able to adaptively focus on the tissue boundary region through the synergy of spatial attention and Laplacian edge enhancement, enhancing the expression of boundary features. By comparing the HTV error map with the BAM-R2UNet error map, it is found that after BAM-R2UNet post-processing, not only is the overall error significantly reduced, but the error improvement in the boundary area is particularly significant, verifying the enhanced ability of the BAM to preserve the organizational boundary.

To further verify the effectiveness of each component in the BAM, a detailed ablation study was conducted on the three parallel branches. Five settings were compared:

(1) Baseline R2UNet (without BAM);
(2) R2UNet + spatial attention only;
(3) R2UNet + channel attention only;
(4) R2UNet + learnable Laplacian edge enhancement only;
(5) Full BAM-R2UNet (spatial + channel + learnable Laplacian).

Quantitative results show that all three branches contribute to performance improvements, and the full three-branch fusion achieves the best RMSE, SSIM, and PSNR. This demonstrates that the spatial attention, channel attention, and learnable Laplacian edge enhancement branches are all necessary and work cooperatively to enhance tissue boundary feature extraction in leg EIT imaging.

To quantitatively evaluate the independent contributions of the BAM and its internal branches, this paper used SNR = 45 dB as the evaluation condition. While keeping the network backbone structure, training data, and loss function unchanged, the ablation model was constructed by successively adding and deleting internal components of the BAM, and the reconstruction performance under each configuration was systematically compared. First, from the perspective of the BAM as a whole, the complete BAM-R2UNet was compared with the baselines without BAM (R2UNet backbone), and a single attention branch was added to the baselines to quantitatively evaluate the effectiveness of the BAM as a whole, as shown in Table 5.

Table 5 shows that introducing any branch of the BAM alone to the baseline (without BAM) leads to performance improvements, with RMSE reduction ranging from 5.5% to 22.8%, SSIM improvement ranging from 6.0% to 10.9%, and PSNR increasing from 0.9 to 1.7 dB. Among these, the edge-enhanced branch contributed the most when used alone (SSIM = 0.7589; PSNR = 19.8 dB), followed by the spatial attention branch (SSIM = 0.7423; PSNR = 19.4 dB), while the channel attention branch contributed the least (SSIM = 0.7256; PSNR = 19.0 dB). The performance of the complete BAM (three-branch fusion) was superior to any single-branch configuration, achieving an SSIM of 0.8329 and a PSNR of 22.3 dB, and reducing the RMSE by 42.8% compared to the baseline, quantitatively confirming the effectiveness of the overall design of the BAM.

**Figure 11:**
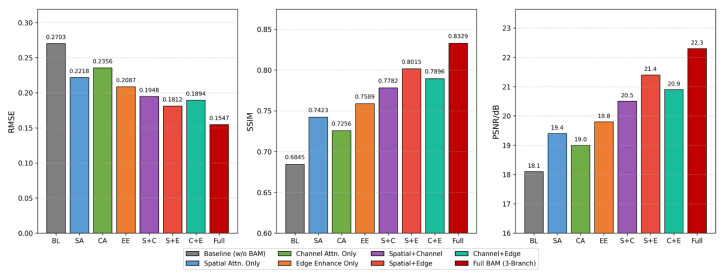
Quantitative comparison of BAM three-branch ablation.

The independent contributions and interactions of the three branches within the BAM—namely, the spatial attention, channel attention, and learnable Laplacian edge enhancement branches—were quantitatively analyzed. Independent ablation experiments for each branch configuration were designed, and the reconstruction performance of single-branch, two-branch, and three-branch configurations was systematically compared. The results are presented in Table 6 and Figure 11.

The contribution patterns of the three branches are summarized in Table 6 and illustrated in Figure 11: (1) In the single-branch configuration, the edge-enhanced branch achieves the best performance (SSIM = 0.7589), confirming the effectiveness of Laplacian edge detection for tissue boundary recovery in EIT images. (2) Any two-branch combination outperforms the single-branch configurations, with the spatial + edge combination achieving the best two-branch performance (SSIM = 0.8015; PSNR = 21.4 dB), indicating a strong complementary effect between the spatial selectivity of the spatial attention branch and the boundary response of the edge enhancement branch. (3) Compared with the optimal two-branch combination, the complete three-branch fusion achieves a 3.9% increase in SSIM, a 0.9 dB increase in PSNR, and a 14.6% decrease in RMSE, indicating that although the channel attention branch contributes relatively little when used alone, it plays an irreplaceable role in feature channel recalibration in multi-branch collaboration. The above quantitative results indicate that the three branches of the BAM are functionally complementary: the spatial attention branch is responsible for identifying important spatial locations, the channel attention branch is responsible for feature channel recalibration, and the edge enhancement branch is responsible for the targeted reinforcement of tissue boundaries. The synergistic optimization of these three branches is achieved through the adaptive fusion of the learnable weights α, β and γ.

Figure 11 compares the three performance metrics—RMSE, SSIM, and PSNR—under eight BAM configurations using a bar chart. From left to right, the metrics show a progressive performance improvement: no BAM → single branch → dual branch → triple branch. The complete BAM configuration leads in all three metrics, confirming the effectiveness of the three-branch parallel fusion design of the BAM.

### 4.6. The Impact of Different Image Resolutions on Reconstruction Accuracy

**Figure 12:**
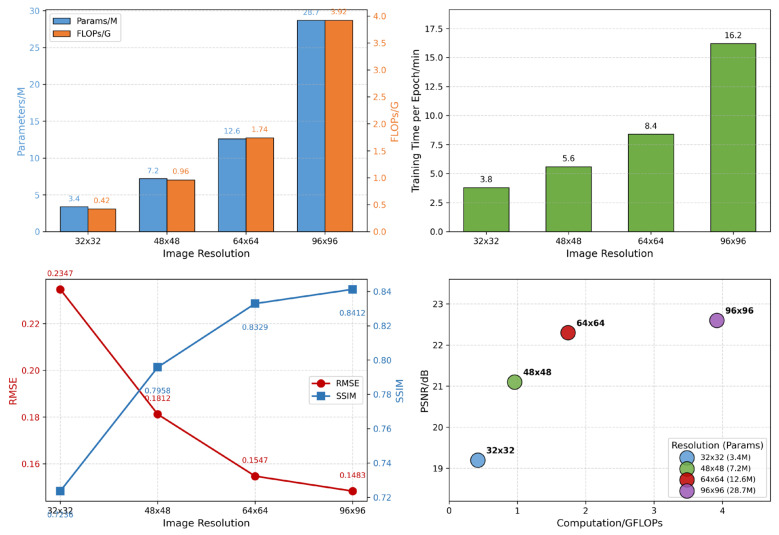
Quantitative comparison of network parameters, computational load, and reconstruction performance at different resolutions.

The resolution of leg EIT reconstructions directly affects the accuracy of tissue presentation and the computational cost of the network. To quantitatively demonstrate the impact of resolution selection on reconstruction accuracy and to justify the use of 64 × 64 resolution in this paper, this section constructs simulation datasets and reconstruction models at four representative resolutions: 32 × 32, 48 × 48, 64 × 64, and 96 × 96. The network structure (BAM-R2UNet), loss function, and training hyperparameters were kept exactly the same, and only the internal feature map size was adaptively adjusted according to the input resolution. This study systematically compared the number of parameters, computational complexity, and reconstruction accuracy at each resolution; the results are shown in Table 7 and Figure 12. The parameter count refers to the total number of trainable parameters of the BAM-R2UNet generator at the corresponding input resolution. FLOPs are measured by the total number of multiply–add operations (MACs) for a single forward pass. Epoch duration refers to the time required to fully traverse the training set once under the same hardware and batch size conditions.

The influence patterns of resolution on reconstruction accuracy and computational cost can be quantitatively observed from Table 7 and Figure 12: (1) When the resolution increases from 32 × 32 to 64 × 64, RMSE decreases from 0.2347 to 0.1547, SSIM increases from 0.7236 to 0.8329, and PSNR increases from 19.2 dB to 22.3 dB. The improvement in reconstruction accuracy is due to the higher resolution providing richer spatial details, which enables the network to more precisely depict the boundary contours of tissues such as skin, fat, muscle, and bone, as well as the morphology of tiny blood vessels and nerves. (2) When the resolution is raised from 64 × 64 to 96 × 96, RMSE is reduced by only 4.1%, SSIM increases by only 0.01 (absolute), and PSNR increases by only 0.3 dB. The marginal improvement in reconstruction accuracy approaches saturation. The number of parameters increases from 12.6M to 28.7M, FLOPs rise from 1.74G to 3.92G, and the epoch duration increases from 8.4 min to 16.2 min. (3) The two lower resolutions, 32 × 32 and 48 × 48, although less computationally costly, have RMSE values 51.7% and 17.1% higher than that at 64 × 64, and SSIM values 13.1% and 4.5% lower than that at 64 × 64, which make it difficult to meet the clinical requirements for fine identification of multi-layer tissues in the legs. Taking into account reconstruction accuracy, parameter count, computational complexity, and training efficiency, the 64 × 64 resolution achieves an optimal balance between reconstruction quality and computational cost and is a reasonable choice for the algorithm in this paper.

Figure 12 illustrates the dual impact of resolution selection on network complexity and reconstruction accuracy across four dimensions: parameter count, computational cost (FLOPs), training time (epoch duration), and reconstruction quality (RMSE/SSIM). These are presented through a parameter/FLOP bar chart, an epoch duration bar chart, an RMSE/SSIM vs. resolution curve, and a PSNR-FLOP bubble chart. The PSNR-FLOP bubble chart (where the bubble area represents the number of parameters) shows that the 64 × 64 resolution lies at the inflection point of the performance–cost curve, where the performance gain from a higher resolution is insufficient to compensate for the increase in computational cost.

## 5. Conclusions

In summary, this paper proposes a post-processing algorithm (BAM-R2UNet) that integrates the boundary attention mechanism to address the problems of blurred boundaries, severe artifacts, and difficulty in accurately identifying multi-layered tissue structures in simulated leg EIT reconstructions. By embedding the boundary attention module in the RecurrentBlock of R2UNet, which consists of three parallel branches—spatial attention, channel attention, and learnable Laplacian edge enhancement—adaptive fusion is carried out through learnable weights to enhance the ability to extract tissue boundary features. The design includes a leg anatomy prior constraint loss function with six constraints—pixel loss, edge loss, hierarchical tissue constraint, total variation regularization, SSIM loss, and histogram matching—to guide the network to output reconstruction results that conform to the concentric multi-layer tissue structure features of the leg from multiple dimensions. Experimental results on the leg multi-tissue simulation dataset showed that: (1) under noise-free conditions, the RMSE of BAM-R2UNet was 0.1523, the SSIM was 0.8376, and the PSNR was 22.5 dB, all significantly superior to the HTV, DnCNN, and U-Net algorithms; (2) under noise conditions with SNR = 30–55 dB, BAM-R2UNet maintained the best performance in all three metrics, especially under strong noise conditions with SNR = 30 dB, when the SSIM value was 114% higher than that of HTV; (3) in the ablation experiment, the introduction of the BAM and the cyclic residual structure increased the SSIM by 21.7% on the U-Net baseline and the PSNR by 4.2 dB; (4) in the BAM attention visualization, the module could adaptively focus on the tissue boundary area. The HTV pre-reconstructed inputs preserve valid EIT physical features, and the two-stage structure combines model-based priors with learning-based optimization to avoid over-reliance on data-driven mapping and ensure physical consistency. This study is restricted to simulation experiments with simplified anatomical models and idealized noise environments, and the proposed algorithm is merely evaluated on a single simulated leg geometry with fixed anatomical priors and a 16-electrode setup. Its generalization to different body compositions, leg sizes, electrode distributions, and other anatomical regions has not been examined, and real-world EIT applications involve more complex anatomical structures and measurement interferences that may influence imaging reconstruction. Accordingly, future work will include physical phantom validation, collection and verification of clinical EIT data, dynamic 3D imaging extension, and model testing on diverse anatomical geometries and electrode configurations to improve robustness and practical applicability.

## Figures and Tables

**Table 1:** Training parameter settings.

Parameters	Value
Image size	64 × 64
Batch size	16
Training cycle/round	120
Generator learning rate	1 × 10^−4^
Discriminator learning rate	4 × 10^−4^
Discriminator training times n_critic	5
Gradient penalty coefficient λgp	10
Basic number of channels	32
Number of cycles t	2
Optimizer	Adam (β_1_ = 0.5, β_2_ = 0.999)
Learning rate scheduling	Cosine annealing

**Table 2:** Reconstruction performance parameters of four algorithms under noise-free conditions.

Algorithms	RMSE	SSIM	PSNR/dB
HTV	0.3812 ± 0.011	0.5347 ± 0.015	14.8 ± 0.4
DnCNN	0.2915 ± 0.009	0.6523 ± 0.013	17.2 ± 0.3
U-Net	0.2647 ± 0.008	0.6891 ± 0.012	18.1 ± 0.3
BAM-R2UNet	0.1523 ± 0.008	0.8376 ± 0.012	22.5 ± 0.3

**Table 3:** Reconstruction performance parameters of four algorithms under noise conditions.

SNR/dB	Metrics	HTV	DnCNN	U-Net	BAM-R2UNet
30	RMSE	0.4536 ± 0.013	0.3847 ± 0.010	0.3512 ± 0.009	0.1892 ± 0.008
30	SSIM	0.3521 ± 0.016	0.4218 ± 0.014	0.4673 ± 0.013	0.7534 ± 0.012
30	PSNR/dB	11.2 ± 0.4	13.8 ± 0.3	14.7 ± 0.3	19.6 ± 0.3
45	RMSE	0.3856 ± 0.011	0.2968 ± 0.009	0.2703 ± 0.008	0.1547 ± 0.008
45	SSIM	0.5289 ± 0.015	0.6478 ± 0.013	0.6845 ± 0.012	0.8329 ± 0.012
45	PSNR/dB	14.6 ± 0.4	17.0 ± 0.3	17.9 ± 0.3	22.3 ± 0.3
55	RMSE	0.3834 ± 0.011	0.2932 ± 0.009	0.2661 ± 0.008	0.1531 ± 0.008
55	SSIM	0.5312 ± 0.015	0.6501 ± 0.013	0.6872 ± 0.012	0.8362 ± 0.012
55	PSNR/dB	14.7 ± 0.3	17.1 ± 0.3	18.0 ± 0.3	22.4 ± 0.3

**Table 4:** Results of ablation experiments (SNR = 45 dB).

Method	RMSE	SSIM	PSNR/dB
HTV (No post-processing)	0.3856	0.5289	14.6
U-Net (Baseline)	0.2703	0.6845	18.1
DnCNN	0.2968	0.6478	17.0
BAM-R2UNet (Complete)	0.1547	0.8329	22.3

**Table 5:** Results of the overall ablation experiment of the BAM (SNR = 45 dB).

SNR/dB Metrics	BAM Component	RMSE	SSIM	PSNR/dB
Baseline (R2UNet, no BAM)	—	0.2703	0.6845	18.1
Baseline + Spatial attention	Only space	0.2218	0.7423	19.4
Baseline + Channel attention	Only channels	0.2356	0.7256	19.0
Baseline + Edge enhancement	Only edges	0.2087	0.7589	19.8
BAM-R2UNet (complete)	Space + channel + edge	0.1547	0.8329	22.3

**Table 6:** Results of BAM three-branch independent ablation experiments (SNR = 45 dB).

Number	Spatial Attention	Channel Attention	Edge Enhancement	RMSE	SSIM	PSNR/dB
#1	×	×	×	0.2703	0.6845	18.1
#2	√	×	×	0.2218	0.7423	19.4
#3	×	√	×	0.2356	0.7256	19.0
#4	×	×	√	0.2087	0.7589	19.8
#5	√	√	×	0.1948	0.7782	20.5
#6	√	×	√	0.1812	0.8015	21.4
#7	×	√	√	0.1894	0.7896	20.9
#8	√	√	√	0.1547	0.8329	22.3

**Table 7:** Comparison of parameters and reconstruction performance of BAM-R2UNet at different resolutions (SNR = 45 dB).

Resolution	Number of Parameters	FLOPs/G	Single-Round Training Time/Min	RMSE	SSIM	PSNR/dB
32 × 32	3.4	0.42	3.8	0.2347	0.7236	19.2
48 × 48	7.2	0.96	5.6	0.1812	0.7958	21.1
64 × 64	12.6	1.74	8.4	0.1547	0.8329	22.3
96 × 96	28.7	3.92	16.2	0.1483	0.8412	22.6

## Data Availability

The simulation code used to generate the data in this study is publicly available at: https://github.com/ZLW917700/bam-r2unet-eit (accessed on 3 May 2026). The generated datasets are available upon reasonable request from the corresponding author due to the large volume of simulation outputs.

## References

[1] Cheng J.K., Sun J.P., Du Y., Liu Z. (1998). Theory and development of electrical impedance tomography technology. J. Beijing Univ. Aeronaut. Astronaut..

[2] Adler A., Holder D. (2021). Electrical Impedance Tomography: Methods, History and Applications.

[3] Miao Z.X. (2020). Simulation Study on the Detection of Moso Bamboo Winter Shoots on Electrical Impedance. Master’s Thesis.

[4] Karhunen K., Seppanen A., Lehikoinen A., Monteiro P.J., Kaipio J.P. (2010). Electrical resistance tomography imaging of concrete. Cem. Concr. Res..

[5] Borcea L. (2002). Electrical impedance tomography. Inverse Probl..

[6] Wang Q., Zhang H., Zhang R., Li X., Wang J., Duan X. EIT image reconstruction method based on DnCNN. Proceedings of the 2021 IEEE International Instrumentation and Measurement Technology Conference.

[7] Li R.Y., Rong Z., Fang T. (2022). Research on Post-processing algorithm of impedance Imaging based on WGAN-gp. Foreign Electron. Meas. Technol..

[8] Ye M.F., Li J. (2025). Post-processing algorithm of electrical impedance tomography based on WGAN-R2UNet. Instrum. Tech. Sens..

[9] Yuan J.J., Rong Z. (2024). Research on post-processing algorithm of tactile electrical impedance tomography based on S-PNet. Instrum. Tech. Sens..

[10] Ye J.A., Tian X., Wang P., Zhang T., Zhang W.R., Liu X.C., Xu C.H., Fu F. (2022). Post-processing model of EIT data based on Multi-branch One-dimensional Convolutional Neural Network. J. Air Force Med. Univ..

[11] Zuo C.L., Li J. (2021). Research on EIT technology based on HTV regularization algorithm. Transducer Microsyst. Technol..

[12] Goodfellow I.J., Pouget-Abadie J., Mirza M., Xu B., Warde-Farley D., Ozair S., Courville A., Bengio Y. (2014). Generative adversarial networks. Commun. ACM.

[13] Arjovsky M., Chintala S., Bottou L. Wasserstein generative adversarial networks. Proceedings of the 34th International Conference on Machine Learning.

[14] Gulrajani I., Ahmed F., Arjovsky M., Dumoulin V., Courville A.C. Improved training of Wasserstein GANs. Proceedings of the 31st International Conference on Neural Information Processing Systems.

[15] Zhang K., Zuo W., Chen Y., Meng D., Zhang L. (2017). Beyond a Gaussian denoiser: Residual learning of deep CNN for image denoising. IEEE Trans. Image Process..

[16] Alom M.Z., Yakopcic C., Taha T.M., Asari V.K. Nuclei segmentation with recurrent residual convolutional neural networks based on U-Net (R2U-Net). Proceedings of the NAECON 2018-IEEE National Aerospace and Electronics Conference.

[17] Ronneberger O., Fischer P., Brox T. U-Net: Convolutional networks for biomedical image segmentation. Proceedings of the Medical Image Computing and Computer-Assisted Intervention-MICCAI 2015.

[18] Wang Z., Bovik A.C., Sheikh H.R., Simoncelli E.P. (2004). Image quality assessment: From error visibility to structural similarity. IEEE Trans. Image Process..

[19] Zhao S.F., Li J. (2022). Electrical impedance tomography algorithm based on two-dimensional convolution neural network. Instrum. Tech. Sens..

